# Use-Case-Oriented Evaluation of Wireless Communication Technologies for Advanced Underground Mining Operations

**DOI:** 10.3390/s23073537

**Published:** 2023-03-28

**Authors:** Marius Theissen, Leonhard Kern, Tobias Hartmann, Elisabeth Clausen

**Affiliations:** Institute for Advanced Mining Technologies (AMT), RWTH Aachen University, 52062 Aachen, Germany

**Keywords:** wireless communication technologies, underground mining, LPWAN, 5G, Wi-Fi, LoRa, advanced mining, mining 4.0

## Abstract

This work aims to give an overview of wireless communication technologies (WCT) for underground applications. Difficulties regarding the harsh mining environment and operational constraints for WCT implementation and use are discussed. Selected technologies are then classified regarding underground mining-specific use cases in advanced mining operations. Use-case-based application categories such as ‘automation and teleoperation’, ‘tracking and tracing’ and ‘Long-Range Underground Monitoring (LUM)’ are defined. The use cases determine requirements for the operational suitability and also quantify evaluation criteria for the evaluation of WCT. The result is a comparison by category of the wireless technologies, which underlines potentials of different technologies for defined use cases, but it can be concluded that the technology always has to be evaluated within the use case and operational constraints.

## 1. Introduction

Mining has always involved the use of new technologies to increase productivity and safety, reduce environmental impacts, and lower operational costs. The industry faces various operational challenges, such as declining ore grades, complex deposit structures, and volatile commodity prices. These objectives are driving factors for digitalization in mining. The vision for digitalization in mining is a connected and autonomous mine with real-time data-based decision-making [1].

The authors of [1] have systematically identified digital key technologies in the mining industry, which can be categorized as Automation and Robotics, IoT, Big Data and Real-Time Data, AI and Machine Learning, 3D Printing, Connected Workers, Drone Technology, Electrification, and Simulation Modelling.

Autonomous driving is for example implemented for long-haulage trucks in surface mining [2] or driverless vehicles underground [3]. Exchanging information on machinery positions is crucial to prevent incidents and optimise processes. Loading processes are not yet automated due to the lack of real-time data regarding, e.g., rock conditions and material positions [2,4].

IoT in mining is one of the basic requirements enabling remote operations and advanced automation [5]. It can be used to gather status information on mobile machines [4], relay data in emergency situations [6] or provide monitoring data on, e.g., rock stability [7].

Analysing (Big) Data with machine learning and AI tools can improve mining operations, for example, in predictive maintenance for conveyor belts [8].

Most of these key technologies rely on the transmission or exchange of (real-time) data, in other words on communication. Communication is a key enabling technology in the automation and digitalisation of mining operations connecting workers, mobile machinery, stationary equipment and mine operation centres. Wireless communication infrastructure must be deployed regardless of the kind of operations to leverage on the technological advancement but is especially difficult in underground mining.

Picking, testing and integrating communication technologies is not only challenging due to the number of possible existing technologies and their technological, physical and legal limits, but also due to the varying mining environments, operational constraints and applications. Mining operations have an enormous amount of variety due to the natural geologic structure and connected mining methods, level of mechanization and automation, as well as size of the operation [9]. In this paper, we will evaluate existing communication applications that range from the transmission of minimal data via ZigBee to the use of digital radio in areas without general infrastructure, to fully remote operations via 5G.

The main drivers for establishing a communication infrastructure that enables advanced mining operations are to (1) improve the efficiency and operations of equipment across the mining value chain, (2) increase safety, (3) reduce costs, and (4) lay the foundation for innovation, all while minimizing (5) environmental impact as an integral part of advanced mining operations [1,10,11].

The aim of this paper is to provide an overview of selected current and potential wireless communication technologies used or suitable for use in mining, as well as existing applications, to assist with selecting and testing the appropriate technology and improving mining operations. Communication networks are already in use in a variety of use cases, enabling different automation and digitalization applications towards interoperability of mine production [11].

**Figure 1:**
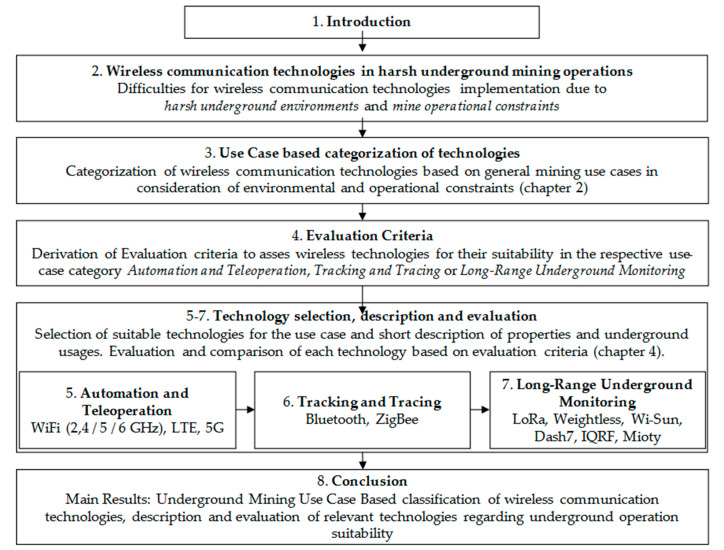
Structure of this paper.

The structure of this paper is summarized in Figure 1.

## 2. Wireless Communication Technologies in Harsh Underground Mining Operations

The successful use of wireless communication technologies in underground operations often faces various difficulties, especially when implementing new technologies.

Based on our experience and research in literature, the following properties have the most significant impact on the underground usability of wireless communication technologies. These properties are derived from environments with very special characteristics and will be discussed in detail in the following section:

1. Shielding of signals due to rock masses [12,13]
2. Underground macroscopic geometry [14]
3. Harsh environmental characteristics [13]
  Mining method, that determines the global mine layout, changes in the environment of the operations as well as connected processes and needs for wireless technologies [12,13,15].

### 2.1. Harsh Underground Environment

Underground mining operations themselves create and take place in confined spaces. Surrounding masses of varying material, often supported by steel or wood installations, are less penetrative for the electromagnetic waves of wireless communication technologies than open spaces. These hinder the transmission of signals significantly and result in a far different signal attenuation when considering different scenarios. Alongside these installed signal hindrances, the signal-based wave modulations and wavelengths that do not penetrate rock masses to a sufficient extent need to propagate along the mining tunnels and drifts. This paper examines the propagation of signals through various scenarios, including straight drift, reflection around corners, and penetration into parallel drifts through dense materials in underground mines. A key distinction in communication systems is the transmission of signals between antennas under line-of-sight (LOS) and non-line-of-sight (NLOS) conditions. Line-of-sight (LOS) refers to the condition where a direct unobstructed path exists between the transmitting and receiving antenna, allowing for the wireless signal to travel directly from the transmitter to the receiver. Non-line-of-sight (NLOS) refers to the condition where there is no direct unobstructed path between the transmitting and receiving antenna, resulting in the wireless signal being blocked or reflected by obstacles such as walls, rocks, and other underground materials before the receiver antenna can be reached. When we talk about NLOS, we consider two subset conditions. We call the first NLOS subset condition ‘Near LOS’. In Near LOS, there is no unobstructed path, but the extent of the obstructions between the antennas is relatively low. The signal can reach the receiver antenna through propagation, such as reflection, diffraction or scattering. The second subset NLOS condition describes situations where signals need to pass obstacles and propagate significantly or even penetrate (rock) material to reach the receiver; we would call it NLOS (as a clear NLOS situation). Both described conditions can lead to weak or no signal reception and increased interference, making it difficult for wireless systems to establish or maintain a stable and reliable connection.

Drift, tunnels and working areas in underground mining offer several distinctive geometric features on a macroscopic scale that are different from other tunnel environments and have a significant effect on the propagation characteristics of wireless radio waves along drifts [12,13]. The propagation along the mining tunnels and drifts creates refraction loss in vertical and horizontal direction, roughness loss of the tunnel surface as well as tilt losses. These are dependent on the following parameters [14]:

− Wavelength,
− Tunnel width, tunnel height,
− Relative permittivity of the rectangular tunnel sidewalls, floor, and ceiling,
− Mine wall roughness,

Long range tilt of the mineral body [15],

− Distance to sidewalls, tilt angle (in radians) about a vertical axis, and transversal positions of the antenna.
− Fortifications in the form of pillars, metal and wooden installation [13].
− Parameter correlations following tilt-variations and tunnel dimensions at a larger scale.

These points lead to increased difficulty in estimating the signal behavior reliably. Experiments to understand the electromagnetic wave propagation have found that wave propagation varies even within the same mine [16].

Underground mine areas are known for their often as harsh defined conditions. This is also connected to the harsh operational characteristics that, beyond the geometric nature of the mine, have an influence on the transmission of signals when using wireless communication technologies. These operational influences can include the following [12,13]:

− Limited LOS (as discussed)
− Waveguide effect at certain frequencies
− Ionized air
− Heat, humidity and their variances along mining sections
− Varying gas concentrations and hazardous gases, fumes, exhaust
− Electromagnetic noise from equipment and infrastructure

The above-mentioned harsh environmental factors can significantly limit the signal transmission reliability. For the operation of underground wireless networks, coverage needs to be ensured. For this, it is crucial to balance between maximizing network characteristics (signal strength, range and other parameters) and minimizing cost and effort [17]. Simulations can be helpful to predict wireless network characteristics in under-ground environments; however, simulation accuracy is limited due to the high level of environment complexity. The authors in [18] show how measuring environmental underground parameters is a challenging and time-consuming task and [19] emphasize the amount and diversity of necessary simulation data needed. Research in this area is ongoing, with projects such as the NexGen SIMS involving the authors and their institute. This project aims to prepare and analyse different communication options in mining environments. However, further research is needed to fully understand the behaviour and potential of these options in the unique and often unknown [20] data situations encountered in mining operations.

### 2.2. Mine Operational Constraints

When going beyond the transmission of signals within the environment, local environment characteristics inherent to the mining operations need to be considered. These determine requirements for the actual physical setup and maintenance of a network and its components and the possible usage, which is highly dependent on the setup within the environment. Mining environments do have an enormous variety depending on the naturally formed ore body and its properties that determine the mining method and used equipment [1]. Mining methods can be classified in various ways. The most relevant for the installation and maintenance of a communication infrastructure are the following factors:

− Repetitive use of areas
− Change of geometry/consistency of roof
− Movement of production/extraction point
− Geometry for signal transmission on a global scale

**Figure 2:**
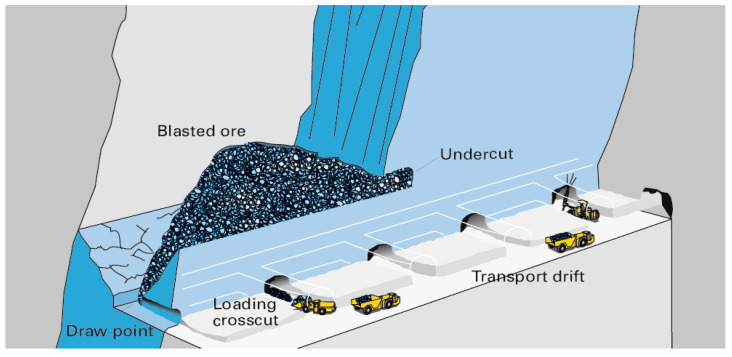
Example of a typical stoping layout [21]. Reprinted/adapted with permission from Ref. [21]. 2023, Epiroc Deutschland GmbH.

Mining layouts that support repetitive and focused usage of the same underground areas with minor change in geometry favour installations of communication infrastructure. Typical examples are most stoping layouts and also caving layouts, where there is a production level with transport drifts connected to draw points that are used excessively and over long periods of time [9,21]. An illustrated layout is shown in Figure 2. Example of a typical stoping layout [21].

Networks can be installed for automation purposes and/or local data transfer and tracing. In these layouts, automation and teleoperation can be more advanced due to the repetitive nature of tasks and the ability to maintain network infrastructure.

**Figure 3:**
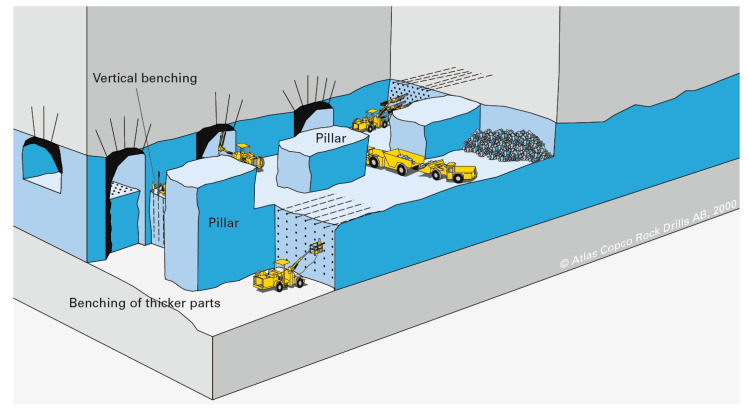
Example of a typical room and pillar layout [11]. Reprinted/adapted with permission from Ref. [11]. 2023, Epiroc Deutschland GmbH.

In orebodies that are mined, it is more challenging to maintain infrastructure in dynamic, constantly moving or expanding layouts because it needs to be located near the extraction point and/or the travel routes of mobile equipment. An exemplary layout for this would be the pillar layout as seen in Figure 3, where there is a lot of interaction of different pieces of mobile equipment at the face and in some cases also a change of the floor due to benching. The extraction point moves constantly or with every drill and blast cycle, which makes it very difficult, tedious and costly to maintain infrastructure that needs to be close to the face.

Additionally, it is critical to consider the sensitivity of devices to blasting impacts, as they may even affect blasting safety. For example, regional or state-wide formulary legislation between the radiated power and a minimum distance to blasting devices and detonators must be taken into account (regional German law: [22] USA, Indiana state law [23]. However, the vast majority of the technologies discussed in this paper only require the minimum distance to be maintained for any radiative power level, which is usually one meter.

Furthermore, established and long-running processes with no changes in used machine type, procedures, etc., are not necessarily time-dependent in their use case, resulting in networks with constant requirements and longer lifecycles than public networks. However, it can be assumed and is probably also feasible that a network can be expanded, updated, or modified with new use cases added in the operation.

## 3. Use-Case-Based Categorisation of Technologies

Aboveground wireless communication technologies are often installed as general infrastructure that can be used by anyone, sometimes connected to provider-based fees. This infrastructure not only exists as public infrastructure but also as local (campus) networks at companies, sites or institutions. This infrastructure is often set up as a multi-layer communication, especially in urban zones:

− LTE to 5G from voice transmission towards high-bandwidth, real-time transmission capabilities
− Public Wi-Fi as a free service, even city-wide
− LPWAN for low-power devices and low data rate applications (e.g., [24,25,26])

**Figure 4:**
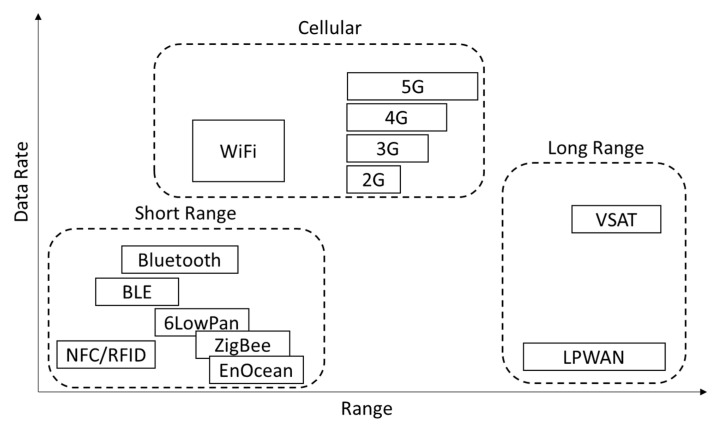
Data rate vs. (aboveground) range of radio communication technologies. Reprinted/adapted with open access permission from Ref. [27].

These listed categories provide connectivity for different use cases and/or devices and vary in their properties. There are various ways to structure wireless communication technologies by properties or application cases. We consider a mapping onto the data rate and range space, which can be seen in Figure 4, as introduced in [27]. Wireless Technologies appear in clusters “short range” (short range & low data rate), “cellular” (medium range & high data rate) and “long range” (long range & low data rate).

### 3.1. Transfering Clusters into an Application-Related Classification

Basic ideas and concepts of a network and its structure can only be partly transferred to underground spaces. Due to the prior described environmental constraints, basic assumptions must be questioned, reviewed or even reconsidered.

The clusters of wireless communication technologies in Figure 4, based on their data rate and range properties for aboveground applications, are only transferable to a limited degree to underground environments. In underground environments, the range of wireless technologies is highly dependent on the specific conditions and is significantly reduced for all technologies. The high signal attenuation underground causes wireless technologies to become more dependent on line-of-sight (LOS) conditions. The further a signal has to travel, and the more obstacles have to be passed, the more it is influenced by the conditions and dependant on propagation properties. This dependency is stronger at higher frequency bands [28], making technologies in the “cellular” and high-frequency technologies in the “short-range” cluster decrease in range even more underground. Low-Power Wide Area Network (LPWAN) technologies, which operate at lower frequency bands, have a weaker LOS dependency, but they still result in a decrease in range underground. In the case of such a transfer, the question of the timeliness of the technology itself for the underground application must be asked. It turns out that timeliness of a technology is linked to its use cases. If a technology fulfils the requirements of a use case, its timeliness is linked to the persistence of the use case itself. Nonetheless, the possibility for a network to be expanded, updated, or modified still stands with new use cases.

**Figure 5:**
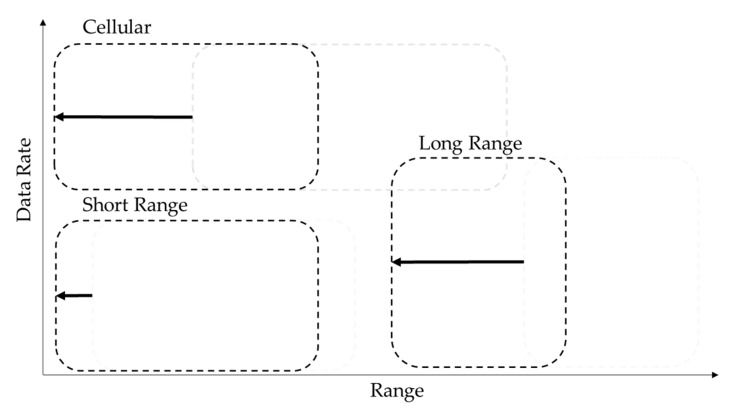
Data rate vs. (underground) range of radio: Shifted Technology Clusters due to harsh underground conditions. The further a signal must travel and propagate, the more it is influenced by the conditions and dependant on its signal propagation properties.

The technology clusters introduced in Figure 4 experience a shift to lower range when deployed in the subsurface environment, which is also illustrated in Figure 5.

Wireless communication technologies in mining are typically used for specific applications (e.g., Wi-Fi for machine supervision and control [29,30]) and not as general-purpose networks, as in urban environments. In alignment with the technology mapping above, we therefore suggest a classification of the wireless technologies in an ordering context, which arranges the technologies in classes of a higher-level mining automation context connected to specific advanced mining operation application cases.

These application cases must be viewed in the frame of individual timeliness of data. The mine operation communication should be realized in real time, but in this context, “real time” is to be understood as “right time”, i.e., the information must be available “in time” for the process [1].

Thus, we suggest mapping the categories as follows:

1. ‘Automation and Teleoperation’

The technologies in this cluster are characterized by high data rates, making them suitable for automation and teleoperation use cases; however, due to the high requirements regarding data rate, they are typically technologically limited to line-of-sight (LOS) and possibly near-line-of-sight usage.

2. ‘Tracking and Tracing’

The technologies in this cluster are characterized by a low data rate and short range, which makes them suitable for tracking and tracing applications, as well as local data transmission.

3. ‘Long-Range Underground Monitoring’

The technologies in this cluster are characterized by low data rates and low power consumption. Early research suggests that they have a high range in harsh underground environments, making them suitable for sensor network applications and potential further underground use-cases.

These use-case-related wireless communication technology classes are now used in the following to define use cases and derive network properties in more detail.

### 3.2. Use Case Examples and Derived Requirements

#### 3.2.1. Automation and Teleoperation

A network for automation and teleoperation covers all use cases around (mobile) machines, their automation, online supervision and teleoperation as control and support by human operators. The automation of a machine is defined as a (partial) takeover of tasks by a machine or automated system given an assigned task from outside and without further or with minimal input from a human. Teleoperation is when the actual command input for the operation performed by a (mobile) machine or system is done at a distance. This approach has the direct consequence of removing on-site operators and maximising safety involved in active operations [1]. Examples of use cases for this category include:

− Supervision of automated loaders or trucks transporting material
− Tele-remote support of loader picking up material
− Supervision and tele-remote combination while drilling
− Robotized ground- or air-based remote inspection of critical areas

In conjunction with these usages, the following data must be transmitted:

− Machine mission, route, action list (down)
− Health state and safety status (up/down)
− Live video and/or point cloud feed (down)
− Control and telemetry commands (down)

A wireless technology must fulfil the following requirements to be appropriate and function effectively for the use cases mentioned above:

− Constant coverage and seamless roaming in working/tele-remote areas
− High-speed connection (~10 Mbit/s) for each machine (possibly higher for VR applications)
− Softly bounding time delays for single way (<1000 ms) for supervision
− Softly bounding time delays round trip (<40 ms) for control (not safety-relevant)

The level of automation and level of human interaction with the machine results in different requirements towards the communication system. Each level of automation can have different requirements [31]. In Ref. [3], the authors consider different levels of automation established for public roads. In Ref. [32], the design of Levels of Mine Navigation Automation (LoMNA) for underground navigation can be found, which also extends the requirements towards communication.

No automation or driver-assisting automation levels resulting in needs for teleoperation set the highest requirements for an installed network, requiring a high data rate coupled with low latency, as the operator becomes part of the control loop. In order for this communication to be seamless and control the machine efficiently, high throughput (e.g., for high-resolution video streams) and low latency in the wireless technology used is required [32,33].

When the level and robustness of automation rises past a certain level, network requirements can be limited by far, even towards a point where there is only a minimal and/or a decentralised network required for the operation of equipment supporting an inter-machine communication and a basic minimum accessibility for higher-level commands (including an emergency stop) [3,31].

Considering the requirements for low latency and high data rates in automation and teleoperation, as well as the clusters shown in Figure 4, the possible network technologies are limited to those in the GHz spectrum, such as 5G or Wi-Fi. These technologies operate in a frequency spectrum that supports high data rates and low latency, but also require wireless installations to have near or direct line-of-sight (LOS) connections between the sender and receiver [34]. Given the constraints of the underground environment, a dense network installation is necessary in order to provide adequate coverage for teleoperation. The network must provide coverage near the mobile machines, as the rock mass limits the range of the network. As the automation and teleoperation areas move or change, it becomes more challenging to maintain the network, as the coverage is very limited in these areas [35].

#### 3.2.2. Tracking and Tracing

Tracking and Tracing applications provide a certain connectivity between machines and towards stationary central points and/or enable traceability of personnel and equipment. The term traceability is defined as “... the ability to trace the history, application or location of an object” [36]. Tracking itself goes a step further. The collected data can be connected to specific machines or workers and is (decentrally) recorded and (centrally) stored. This provides a temporal and spatial information density that can yield new insights when analysed.

Such network can be installed at points of interests such as intersections, entry/exit points, infrastructure areas, loading/dumping points, on mobile machines and infrastructure. This sort of “network” does not provide full connectivity or coverage, but very local connectivity that can transmit information to central units when connected to a central infrastructure at all. These can provide actual transmission of data sets or provide information towards the mobile machine and driver or towards a central control unit. Examples of use cases for this category include:

− Transmission of locations (tracing for vehicle or central control)
− Transmission/reading of ore pick-up/drop-off when passing
− Pick-up/drop-off of collected sensed data from sensors or machines at a local scale
− Passing of sensor data in direct surroundings
− Detection of approaching vehicle or worker
− Tracing of worker and equipment towards central units (when passing)

In conjunction with these usages, the following data must be transmitted:

− Position or area
− Quality, quantity and distribution information of materials
− Various machine performance or environmental parameters
− Information of existence of unit/worker within certain range or area
− Collision perceptions
− Other collected datapoints

A wireless technology must fulfil the following requirements in order to be appropriate and function effectively for the use cases mentioned above. The requirements are as varying as the use cases:

− High (Mbit/s) or very low data throughput (Kbits/s)
− Coverage within single or precisely distributed defined connectivity area(s)
− Robust (i.e., transmission detection)
− Quick connection setup and transmission
− Robust and sufficient for safety-relevant applications, incl. compliance
− Battery lifetime of remote sender/receiver

#### 3.2.3. Long-Range Underground Monitoring

Underground monitoring applications, such as IoT sensor networks, are characterized by low power consumption and long range. LPWAN technologies meet these requirements, but the wireless technologies are also characterized by a low data rate. LPWAN applications can be found in many fields, such as asset tracking, smart city applications, smart metering and smart agriculture [27].

In the mining sector, LPWAN technologies have a great potential due to increased need for connectivity and the potential of an increased range in underground spaces compared to existing technologies. These properties could enable data transmission with sparse installations in dynamic and widespread areas. Research underground is limited, but first investigations and tests (e.g., [37,38,39]) show a promising application in the underground area.

Use case example are:

− Ubiquitous coverage for safety
− Transmission of health/process status and/or position of worker mobile equipment
− Environmental monitoring of rock stress, gas concentration, etc.
− Asset tracking
− Predictive maintenance
− Supervision of certain areas

In conjunction with these usages, the following data must be transmitted:

− Health/process status and position
− Environmental data
− Single (status) images
− Operational or process data

A wireless technology must fulfil the following requirements to be appropriate and function effectively for the use cases mentioned above:

− Softly bounding time delays for a single path (<1000 ms) for supervision (single point-to-point connection)
− Support of numerous devices
− Very low-frequency (from per minute to days) transmission of data
− Not dependent on electric infrastructure; sufficiently high battery lifetime of extending stations and sensors
− Very wide and penetrating coverage of single stations

### 3.3. Conclusions

The network types, use cases and resulting requirements are now directly connected to technologies. These can now be mapped onto the data rate and range space, which to some extent also aligns with acceptable cost and the ability to adapt to changing environmental conditions. Each axis has a gap between the technologies. The most notable gap can be seen in Figure 4, which shows that there is no technology that offers both a high data rate and a high range–this is due to the lack of penetration.

**Figure 6:**
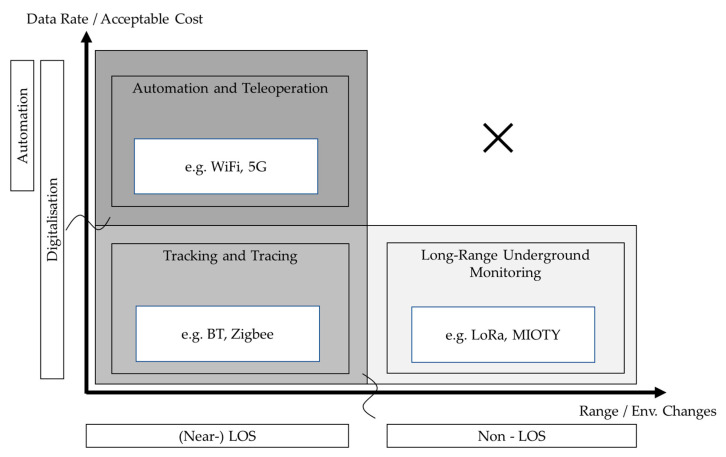
Mapping of basic network properties, use cases and connected technologies.

According to the requirements from the use cases, automation and teleoperation networks can serve digitalization applications, while tracking and tracing as well as Long-Range Underground Monitoring technologies can offer lower data rate connectivity at potentially lower costs and with less infrastructure installation, making them suitable for a wider range of applications. Each network type connected to technologies creates a space supporting the different use cases (Figure 6).

## 4. Technology Evaluation Criteria

Three different kinds of networks supporting different use case spaces have been identified in the previous section. They have different limitations connected to the aligning of communication technologies in combination with the constraints of an underground environment. Various communication technologies exist, and many are available on the market, but only a very limited few have proven use cases in mining applications. Communication networks and their establishment in particular use cases face a multidimensional problem of weighting and correctly assessing the requirements.

The classification, analysis, and potential selection of technologies for communication require a consistent criterion and schemes. The following section provides technical and non-technical requirements for communication technologies.

The framework for the analysis is based on the framework introduced in [40] and includes the following criteria:

The market situational analysis is expressed in terms of market maturity, approval and cost. On a concrete physical layer, one speaks of the required infrastructure in the application area, the forms of the infrastructure in relation to the need, their service availability and the intrusiveness of the same. Evaluation of the network structure itself is expressed through the points ‘coverage’, ‘scalability’, and ‘number of users’; and further, ‘update rate’, ‘output data’ and ‘robustness specifications’ of wireless systems. Specific to the use of a positioning system for workers is the group of characteristics of accuracy and precision*,* as well as privacy.

To use the framework in our work, we propose some modifications by combining, expanding, and/or eliminating criteria from it. We will provide further explanations on the modified criteria and incorporate scales, so the framework can be systematically applied to classify technologies based on the criteria. The criteria of accuracy and precision are not being considered, as they are specific to localization/positioning. Additionally, the criterion of privacy is not relevant in this paper, as it pertains to encryption and security protocols, which are beyond the scope of this research, as they are not fundamentally different from those of surface applications when considered in the context of underground operations.

The technical security of a network is certainly a point of discussion for networks in general and of interest for mining operations. Examples such as IOT Goes Nuclear [41] show the impact and vulnerability that networks of ideal density can have. Due to the physical separation of the underground network on top of the software security, the total security is immensely improved. However, it must be noted that the safety of the network also depends on the safety of the possibly connected networks that reach to the surface and global connectivity.

The other criteria have been adapted as follows:

### 4.1. Market Maturity

Given definition:

The market maturity is defined as the current development maturity of the proposed technology and can be categorized into three market maturity levels: concept, prototype and product.

Additional commentary:

Even though mining operations are highly innovative, the technology readiness in terms of communication networks lags behind in these environments [40]. Outside of mining, at a product level, technologies have often barely been tested in the mining environment for various reasons.

Suggested scale:

We propose to use the Technology Readiness Levels (TRL) [42] to indicate the maturity

1. TRL 3 to 4: Proven in concept, in lab, or another environment.
2. TRL 5 to 6: Validation to demonstration in relevant environment.
3. TRL 7 to 9: Demonstration to actual system in operational environment.

### 4.2. Approval

Given definition:

Assessment of the legal basis for the application of a specific technology with certain peculiarities and specific functions. It must be verified, for example, whether a technology operates on frequencies subject to legal use regulations that conflict with underground applications.

Additional commentary:

A legal and safety-compliant use of a technology and frequency band must be given. Furthermore, the use of a certain band is connected to licensing of usage. Due to legislative differences at the national level, which apply to most bands used for RF technologies, this is an issue that can affect a product for specific applications and must be considered.

Suggested scale:

As a scale, we propose considering the availability regarding legal compliance and licensing needs.

1. Approve bounded to certain license-holders
2. Approvable by application by operator/supplier
3. Open usage within legal limit by anyone

### 4.3. Required Infrastructure and Intrusiveness

Given definition:

Required infrastructure refers to the hardware that needs to be installed to perform the communication tasks at hand. Intrusiveness refers to the degree to which the communication system interferes with personal or workflow activities.

Additional commentary:

The infrastructure is of central importance to differentiate the technologies under consideration. Technology options can be distinguished by the need for hardware installed within the environment to be connected at mobile or stationary systems. This contains possibly needed (wireless) network connection infrastructure and a permanent power supply. The introduction of some sort of communication is meant to have a positive change on the workflow and or process, but installation, reinstallation, and maintenance can be intrusive to existing processes and is in this case combined with the needed infrastructure.

Suggested scale:

Needed infrastructure and connected intrusiveness:

1. Dense installations connected to (cable-based) network and power supply
2. Sparse installations connected to (cable-based) network and power supply
3. Decentralized installations without power supply (battery-powered)

### 4.4. Coverage and Robustness

Given definition:

Coverage refers to the spatial extent to which the system’s performance must be guaranteed, while robustness is defined as the resistance to disturbances, including temporary or permanent network failures.

Additional commentary:

Coverage and robustness of signal propagation and possible non-LOS signal propagation and even massive rock penetration must be considered at different levels. This is connected to the density of needed installations and robustness of coverage of new or distant areas. Extendable coverage with additional hardware infrastructure must be given (under consideration of data transmission capabilities).

Suggested scale:

We suggest differentiating signal transmission capability of signals under various conditions regarding LOS, harsh environment and penetration of rock or rock masses.

1. Sensitive to (direct) line-of-sight (LOS)
2. Near LOS (signal transmission tens to hundreds of metres), insensitive to harsh conditions (e.g., dust and humidity)
3. NLOS, propagation of signal (signal transmission 100th to 1000th of meters) or even penetration of rock or even rock masses (signal penetration 10th to 100th of meters)

### 4.5. Scalability and Number of Users

Given definition:

Ability of the system to increase its coverage area and the number of users/devices the system can support.

Additional commentary:

Within the changing and expanding environment as well as rise in applications, the extension in space and number of users shall be considered in combination.

Suggested scale:

As a scale, we propose the (dynamic) expendability with no or limited additional installations and network capabilities to maintain data rate per device.

1. Expansion connected to deployment of hardware and/or infrastructure with centralized hub, lowering possible data rate per device and user
2. Expansion connected to deployment of hardware and/or infrastructure with multiple hubs expanding the network
3. Self-expansion with mesh functionality of network by decentralized nodes, rarely lowering data rate per device and user

### 4.6. Data Rate and Network Delay

Given definition:

The data rate describes the throughput a given wireless technology can offer. Latency or network delay describes the time a bit of information needs to get from one communication endpoint to the other communication endpoint.

Suggested scale:

The higher the communication requirements of an application, the higher the requirements for large throughput and low latency. We therefore propose a scale from low to high, where throughputs in the range of bit/s to kbit/s and latencies in the range of up to one second are acceptable (category ‘low’–e.g., applications for sensor data acquisition) up to throughputs in the order of Mbit/s and latencies in the range of milliseconds are required (category ‘high’ e.g., teleoperation).

1. low ~bit/s–kbit/s, single seconds
2. medium kbit/s–Mbit/s, tenth of a second
3. high ~10 Mbit/s or possibly higher, milliseconds

## 5. Automation and Teleoperation

### 5.1. Technology Selection

In the context of automation and teleoperation, the need for low latency and high data rates (as discussed in Section 3) limits the feasible network technologies to those in the GHz spectrum, such as 5G, LTE (Long-Term Evolution) standard/4G, as well as Wi-Fi 2.4/5/6 GHz. As they require (near) line-of-sight (LOS) conditions to maintain signal strength in the face of high attenuation in that frequency spectrum, feasible areas of usage would be repetitively used areas where infrastructure can be installed and maintained. These areas must cover (in a relatively small area) full process usage cycles of machines that then can be monitored or teleoperated (e.g., a full haulage cycle).

### 5.2. Technology Description

#### 5.2.1. Wi-Fi

The wireless fidelity (Wi-Fi) is a set of networking protocols. It is the trademarked and most used standard for devices in a wireless local area network (WLAN). The Wi-Fi 5 standard introduced in 2014 was succeeded by Wi-Fi 6 (2019) and now Wi-Fi 6E in 2020 [43]. The main difference between the standards is their accessible GHz spectrum. WiFi 6E is the extension of the 2.4 GHz and 5 GHz spectrum to include the 6 GHz frequencies to be used in an unlicensed manner [43].

Wi-Fi 6E operates in the 6 GHz band from 5.925 to 7.125 GHz, 1200 MHz from 5.925 to 7.125 GHz having been allocated for Wi-Fi applications. Previously, Wi-Fi standards (including Wi-Fi 6) used the 2.4 GHz band (2400 to 2495 MHz) and the 5 GHz band (5170 to 5835 MHz) [43]. This new band allows Wi-Fi 6E to operate with 14 additional 80 MHz channels and seven additional 160 MHz channels. This talk about channels provides a quantifiable term for areas without crosstalk. This is an often-cited problem in dense urban apartment areas with each household having a router. In numbers, the old 2.4 GHz standard could have three 20 MHz channels or combine them via multi-channelling to a single 60 MHz channel [44]. 5 GHz units could use all of 19 channels, with 20 MHz combining them to up to two 160 MHz channels [44]. With 6E, even 24 channels are possible, although the upper limit in the frequency and its usage varies heavily for different nations or regions [44]. On one 160 MHz channel, multi-Gbps throughput is possible [43]. Therefore, Wi-Fi 6E can have three separate maximal bandwidth operations at the same time if it uses the full spectrum and therefore occupies the entire wireless communication spectrum aside from millimetre waves from 5G discussed later.

The improvements in the protocols in combination with the possibility to use the large number of possible channels in all three spectra set of Wi-Fi, simultaneously, is a huge advancement for the Wi-Fi technology in terms of the available number of usable channels, resulting in the potential bandwidth and number of users [45].

For these reasons, Wi-Fi 6 has been considered an alternative to a (private) 5G cellular network for communication and data exchange in coal mines [35]. Wi-Fi has a different set of characteristics stemming from its targeted indoor application compared to cellular networks, namely, the lower coverage area per basis and the lower power consumption [46]. While it has been calculated that the 5G infrastructure can be replaced in a coal mine with Wi-Fi 6, one more aspect of how the Wi-Fi relevant bands are structured can be analysed [16].

Wi-Fi 5, 6 and 6E had a big focus on increasing the number of channels per band, which a single unit like a home router could send and receive data on. This is of significance, as all routers in proximity to each other must share this finite number of channels. This can happen quite fast in, for example, apartment complexes with each household owning a dedicated router [1,43].

Mining operations do not suffer from these problems, as the routers are placed for efficiency and urban signals are minimal or zero. This unique situation could enable the usage of multiple bands simultaneously for a great data rate improvement. This is already done by some router models but only on the scale of a few channels [47].

An important aspect of the discussion about the new devices with WiFi 6 and higher is the connection between devices on the higher frequency bands, which are less robust in harsh conditions and over greater distances. When a connection at 5/6 GHz can only be partially maintained, the network will only be established in that section or situation on the more robust 2.4 GHz band [48]. This results in a reduction in bandwidth, higher latency and fewer possible users–potentially not fulfilling needed network specifications under some circumstances, since the network is not managed or prioritized. However, the 2.4 GHz range is greater and the signal has greater propagation or even penetration [49]. Thus, a reduced functionality of the network can still be maintained with 2.4 GHz.

One of the standard applications is the supervision and teleoperation of LHDs underground. The Wi-Fi 2.4 GHz network is mostly standard, having 10 Mbit/s available per machine and 100 ms maximum delay (incl. roaming). It is also noted that the used Wi-Fi is a limiting factor in the number of parallel running machines. An update to later Wi-Fi standards enhances network capabilities that have a direct effect on usability and thus productivity [30,50]. Prior noted risks remain, especially when the use of the network is extended for further applications.

A specific application of the Wi-Fi Network is the implementation of a so-called “Mesh network”, which is a part of IEEE 802.11s [51]. In the ARTUS project [52], a mesh network was implemented based on 2.4 GHz Wi-Fi routers, with the goal being to enable machine-to-machine communication and dynamic expansion of a locally required network based on the Wi-Fi IEEE802.11s standard [51]. In a mesh network, all primary units act as routers that generate their own Wi-Fi signal and have a transmission function for data between routers, similar to regular Wi-Fi repeaters. This enables the routers or “mesh nodes” to connect to each other and potentially to a stationary modem for internet access, allowing for a completely linked network where any two points or nodes are connected through a path that may include intermediate nodes [53].

Via path optimization algorithm, it is possible to have a self-expanding and dynamic network. This can be achieved with a combination of stationery and machine-mounted nodes. The concrete use case of this Wi-Fi Option and configuration is the machine-based dynamic network in open pit and underground mines [52].

Wi-Fi has a great potential for mining applications and is already in wide use. Nevertheless, the networks always seem limited to a single use case and are always “best-effort” unmanaged networks, that still can serve the purpose.

#### 5.2.2. LTE and 5G Cellular Networks

Cellular networking is a fundamental backbone of various mining operations. The deployment of the fifth generation of the “Long Term Evolution” (LTE) for telecommunication is called 5G. It offers improved data rates from the 4G LTE’s 100 Mbit/s up to 10 Gbit/s for 5G. Furthermore, the new standard reduces the latency to a level of one to a few milliseconds. This allows for real-time remote-controlled units, which function as a major incentive for industrial applications [11].

This 5th Gen standard for broadband cellular network began installation worldwide in 2019 [54]. Prior to 2019, the mobile network standard consisted mainly of 3G and 4G, with 4G Advanced added in 2010. As the name “Long Term Evolution” suggests, it was set as a standard to overlap the use of 3G and 4G, which also led to an adjustment of the 4G standard [55]. However, it is common to just refer to LTE as the precursor to 5G. Backward compatibility is included in the 5G standard, extending the use and spectrum range for mobile network devices. These hybrid wireless networking systems separate the standards in their specified applications. 4G is sufficient for voice calls and (relatively in this use case) low data transmission. This frequent and semi-irregular workload for the network can take advantage of the longer range or distance between the network basis of the 4G compared to the 5G.

The successive approach of 5G in terms of use and maintenance of the bands, i.e., the frequencies used by the predecessors 4G and 3G, becomes very clear when talking about the range bands that have been allocated and designated for 5G [56]. The most used terms for the division of the spectrum used and its effective characteristics and application areas are low-band 5G, mid-band 5G and high-band 5G.

Low-band, sometimes called “Nationwide 5G” (by Verizon) or “Extended Range 5G” (by T-Mobile), is used to combine and preserve old 1G and 2G, as well as unused spectrum in the 600 MHz to 1 GHz range with an estimated 10 to 20 Mbit/s with the 5G standard. The exact frequencies used always depend on national regulations [57], but there is also the term Ultra High Frequency (UHF) 300 MHz–3 GHz [56] by the International Telecommunication Union (ITU) in general. These decimetre radio waves propagate mainly in a straight line and are reflected by smooth obstructions like walls whose surface satisfies the Rayleigh criterion [58,59].

Mid-band 5G ranges from 1 GHz to 6 GHz, but it is more widely considered to be in the 2.4 GHz to 4 GHz range. Here is where the well-known C-band for satellite communication lies [60], from 3.7 GHz to 3.98 GHz. It can cover large areas and provide fast speeds ranging from 300 Mbps to 1 Gbps [61]. It combines speed, range, penetration, and capacity. The main application for mid-band 5G will be in suburbs and cities where demand is significantly high.

High-band 5G spans new frequencies made available in the millimetre wave spectrum. It ranges from 24 to 39 GHz. Here the largest gains in terms of bandwidth and latencies can be found. It provides 1 Gbps speeds but can reach up to 10 Gbps [54] under optimal conditions. Latency can be as low as 1 ms [54], but all this only with lower ranges. Waves with millimetre wavelength can only travel short distances and do not penetrate walls [60]. Waves are easily jammed. To relay these signals inside buildings, whether for signal boosting or 5G internet, 5G antennas and equipment will be needed.

Development of applications in mining in the low band is difficult due to needed viable network properties conflicting with local regulations that apply even underground. At this point of technology development and 5G adoption, the mid-band is being utilized for first-use cases in underground spaces. The high-band (that has fantastic performance parameters) lacks robustness, in the understanding of the authors, for the application in harsh and changing underground environments, especially because the properties would be most valuable for remote control at the face.

Currently, combinations of 4G LTE and mid-band 5G-based networks can be found, for example, at coal mines in China [34] as well as in current research activities at underground test facilities in Kvantrop, Sweden. Various use cases exist/are being tested, such as teleoperation, fleet control, drone-based remote inspections and mine ventilation.

Another aspect to consider is the use of 5G as a private 5G network. It describes a local area network for dedicated wireless connectivity within a specific area alongside provider licensing having a frequency slice allocated for a specific application [62]. Specific management of the network can be applied to a 5G private network. This includes data periodization and assured latency for certain applications that can be a key factor of the technology and a necessity for safe and efficient teleoperation especially in high-density areas and in the advancing use of connectivity in mining. Furthermore, enterprise users have the ability to define their own data policies and keep sensitive and proprietary information local [63].

Another aspect to consider is a private 5G network. It describes a local area network for dedicated wireless connectivity within a specific area [62]. That leaves the opportunity, regardless of provider licensing, to utilize the network for industrial purposes. A 5G private network can be managed; enterprise users also have the ability to define their own data policies and keep data local [63].

**Figure 7:**
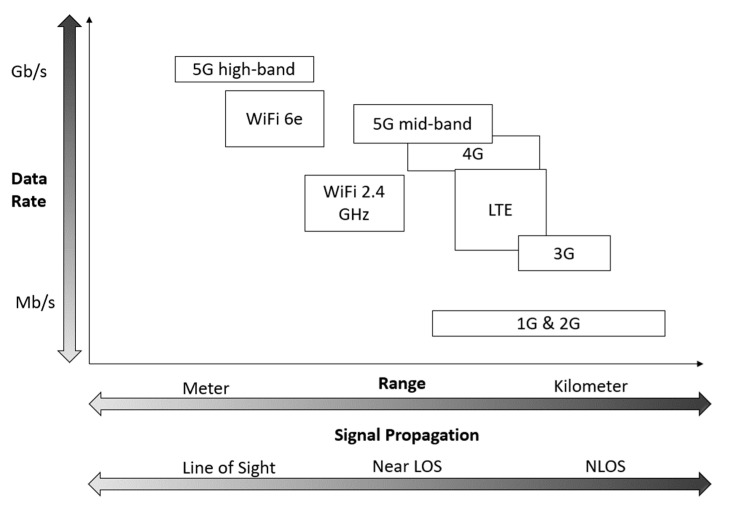
Generalized and simplified visualisation of the Wi-Fi, 5G and other cellular technologies.

In summary, 5G is suitable for a fully teleoperated or autonomous mining fleet from the specification side; however, the feasibility of the hardware and application in the field of an active mine still needs to be researched. Furthermore, it has the potential to be a WCT that can leverage other technologies (Figure 7).

### 5.3. Classification of Technologies by Parameter

1. Market Maturity/TRL

Both Wi-Fi with the 6E standard and 5G have a market maturity of products. However, it is difficult to evaluate both in the context of underground mining. Both technologies are explored for their application in active mines [64], whereas LTE and Wi-Fi 2.4 GHz have already been adapted and used in various use cases.

2. Approval

It turns out that Wi-Fi, but even more so 5G, have specific requirements and regulatory constraints due to their respective bandwidths. While both technologies are generally well recognised and regulated in terms of their use of the bands, most laws are designed for public or private use. This also includes the need to ensure that non-public communication protocols operate on the chosen frequencies and have reservations on the use of the bands for safety reasons.

In addition, there are state-based regulations, such as the radiated power, which must be observed when dealing with electrical detonators for blasts in mines (e.g., [22,23]). Extensive tests and applications are required before regulations can be exceeded due to the shielding of the walls of a mine. Furthermore, 5G and its operation is only possible through the license carriers, whereas Wi-Fi is available even at 6E from every consumer in its hardware configuration.

3. Required Infrastructure and Intrusiveness

The effective maintenance of a network is only possible with a permanent connection to a power supply; battery-powered systems are not feasible. Further, a cable-based communication infrastructure is needed (except for mesh applications). Compared to older LTE standards, 5G has increased consumption [46,54]. All WCT have an intense need of infrastructure and are intrusive.

4. Coverage and Robustness

For 5G, there is a lack of long-term data on the spread of signaling in mines in active operation and of different types. Therefore, a quantitative assessment is not possible at this time. Measurements in urban environments can give an order of magnitude, but only under the condition that the reflective effects of the mine walls are negligible, which is not possible without data. With Wi-Fi, the situation is different. There, a Near LOS setup could be achieved at the applied scale [16].

5. Scalability and Number of Users

The coverage of the networks is only possible without data rate loss by expanding the number of gateways. However, the functionality of the multi-channel is lost if the density of users on Wi-Fi is too high, especially with 2.4 GHz. The 5G standard has such a high number of possible supported users at the same time and over the same area that even high-traffic and active sections of a mine do not provide a problem.

6. Data Rate/Network performance

The technologies are applicable for teleoperation and meet the best score on the scale with high ~10 Mbit/s rates and milliseconds in latency [35]. However, 5G and 6 GHz Wi-Fi do have an additional potential to support higher rates (indicated by a +).

The table below summarizes the technologies and their classification in each evaluation criterion introduced in Section 4. The scales for each criterion consist of three ordered labels, starting with the ‘worst’ and ending with the ‘best’ label. For better recognition, the labels are marked with colours in the Table 1, ranging from red (worst) to green (best) for each category.

### 5.4. Conclusions

The introduction of teleoperation and automation in the mining sector offers numerous possibilities, but the choice of options for implementing the necessary networking structure is limited. From among the feasible network technologies, 5G and Wi-Fi 2.4/5/6 GHz are expected to be able to deploy a network with appropriate characteristics (see [16,65]). Feasible areas of usage would be repetitively used areas where infrastructure can be installed and maintained. However, it is important to note that 5G and WiFi 6 Ghz have not been extensively tested underground yet, and further research is needed to evaluate their operational capabilities in those environments. Additionally, another important factor to consider is the access and licensing of the hardware. 5G components are not as readily available from a variety of vendors as Wi-Fi hardware. On the other hand, 5G offers the opportunity for a managed and controlled network, resulting in reliable communication links having a higher quality of service.

## 6. Tracking and Tracing

### 6.1. Technology Selection

In the technology selection process, Bluetooth and Zigbee have been selected as the most popular technologies with a wide range of applications. While there are other technologies that could be considered, such as Zigbee Pro, NFC, WirelessHART, and EnOcean. Bluetooth and Zigbee have a proven track record with established use cases underground (e.g., [66,67,68,69]) and are therefore described in more detail in the following section.

### 6.2. Technology Description

#### 6.2.1. Bluetooth

Bluetooth was introduced in 1998 and, via continuous development and version control, grew to one of the most used communication methods in commercial and industrial applications. The technology is based on ultra-high frequency radio waves in the ISM bands, meaning 2.402 to 2.48 GHz. Bluetooth gained this high status as the Bluetooth Special Interest Group (SIG) improved the specifications, such as data rates, signal reach and protocol features, including low-energy transmission and direct beacon pairing. These upgrades in the form of Bluetooth versions with backwards compatibility allowed the technology to be kept in the fast-evolving field of commercial electronics. The resulting refinement of this communication option is a key aspect alongside the general specifications for its use in mining [70].

This size and market representation make the technology extremely interesting for the integration of new systems into the Internet of Things or into local area networks. When this technology is considered in the context of tracking and tracing, there are a number of opportunities that arise even in the harsh environment in underground mines [66,71].

Proximity warning systems (PWS) have already been mentioned as one of the tracing cassettes. This application is intended to signal an imminent collision between the nodes and thus their carriers via mounted or carried network nodes. In [66,67,68,69], a PWS network by means of Bluetooth beacons in dedicated devices on people and machines is tested. The test series in the Yeonhwa underground tunnel was considered conclusive and successful.

The scientists in [72] set up a Bluetooth beacon-based underground navigation system, a so-called BBUNS, in a limestone mine. This is an advantage of a mine section equipped with tracing and tracking, as it leads to the analysis and optimization of operational processes. The route optimization and the decision on the route is applicable in the three forms of mining cycle, human-driven teleoperation, and automated conveyance. This information is important in all three situations, and the beacon network and data analysis are used to reproduce an ideal line on the display of the driver. Cost and implementation proved to be practical and robust. Precision and accuracy were also sufficient for the critical situations of a PWS. Due to the large number of mobile nodes on machines and workers, the coverage is given in the concept of Dynamic Coverage. This coverage can be implemented with mesh-type networks, as described in [73]. In this paper, the researchers have developed a monitoring system based on a portable Bluetooth device. It is used for health monitoring and tracking in a standard working situation or in an extended emergency mode in the event of an accident. Data is collected and transmitted through a mesh network of the devices themselves.

Still to be considered are the technical parameters of the data rate, data type and range, which are clear from the experiments for a sufficient application at local medium to short ranges [66,67,68,69].

Bluetooth is an extremely widespread and developing wireless communication standard that is already being used successfully in various applications for local data transmission, tracking and tracing, which is also finding its way into application in advanced mining operations.

#### 6.2.2. ZigBee

ZigBee is a communication option created and maintained by the ZigBee Alliance. It works around 900 MHz with one channel and around 2.4 GHz with up to sixteen channels. The technology has a low data rate and power consumption. An often-cited feature is the direct and specialized implementation of a mesh network structure (see Wi-Fi). These features allowed the technology to enter the market for smart home automation and expansion. Developed by the ZigBee Alliance based on IEEE 802.15.4, there are three basic module types in a ZigBee network. These modules generate the private, low-data, and short-range network (20 m to 100 m) [74]. The coordinator device establishes and handles the communication and paths between routers. They are arranging and managing the mesh structure. Therefore, these modules can only be connected to other ZigBee modules of the coordinator type. The ZigBee Router manages the actual data exchange between all three modules and forms vertices between the other two modules. The data rates are in the region of 20–250 kbps [75].

Modules are capable of low-power sleep mode and can be used with over two hundred other units in one network. This has been shown via implementations of ZigBee networks for gas concentration monitoring [76,77] as well as for tracking miners in dangerous areas for increased safety and accident alerts [78].

Safety-critical environmental factors have a primarily three-part structure [79]. Due to the high mobility of the receiver/transmitter unit, which results from low weight, low power consumption and mesh coupling, it is possible to enable network-based stationary data acquisition underground, mobile workers/machines and data centres [79].

Sensor units that collect data such as temperature, air quality and other environmental factors are located at fixed and critical points of operation in the underground mine. These obtain a temporary, on-the-spot evaluation and, if necessary, send an alarm event to workers and machines in the immediate vicinity of the network or of the sensor itself. Central monitoring units can record the data flow of sensors linked to central/active positions, monitor them over longer periods of time and link them to other measurement units.

ZigBee was developed as a smart home communication protocol with dedicated hardware and a relatively short range of m to 100 m. However, this simple quadratic relationship for extensions in the dimension of length no longer applies in NLOS situations. This is the case, for example, in underground mines, where pillars or simply walls usually represent a total signal blockade. Due to the higher number of nodes in the same area, the necessary infrastructure is denser. However, this also provides the opportunity to implement an emergency tracking method for workers in dangerous sections of the mine, having a resolution instead of only a “check-in” tracking. One would know via the network the last closest node from which the signal of the mobile unit was received [76,77,80]. The execution of a rescue plan would be faster as “in case of accidents or miners being trapped underground, the tracking unit [ZigBee] will help in locating the real time location of the miner” [78].

It is conceivable to add a ZigBee connection to existing measuring and monitoring sensors. This network would be able to be implemented without interaction with existing networks. The low hardware volume required for a ZigBee deployment brings with it the possibility of being deployed in adverse mine sections [81].

ZigBee as a low-power mesh network, having a local range and option of twenty to one hundred meters, and therefore is highly suited for selective monitoring tasks in active mining environments. It is in use for different tracking and tracking use cases for locating workers and assets and can be utilized to create local low-power mesh networks but has not found wider adoption.

### 6.3. Classification of Technologies by Parameter

7. Market Maturity/TRL
8. Bluetooth has been found in a variety of consumer devices for decades. Not only does Bluetooth find a place in communication between machines and collision detection, but this application of Bluetooth in machine communication has even found research work in mining environments [72,82]. ZigBee is a well-known technology that can be found in many smart home products. Thus, a clear TRL of 9 can be seen in the public market; however, with the special focus on the application in the mining environment, it looks different. The technology and concept of ZigBee have research potential. They have inherent features such as the recursive update possibility [41], which can be analysed in terms of mining applications. However, these considerations have not yet made it to product-based implementations for mining.
9. Approval

Due to the commercial spread of consumer products and the microwave frequencies used in the technologies, their use is fundamentally justifiable and not subject to any special requirements.

10. Required Infrastructure and Intrusiveness

The low power consumption of the technologies makes it possible to model off-grid node distribution. Of note is the emergence of sleep mode protocols in Bluetooth enabling the possibility of battery use [70].

A big advantage of Bluetooth and Zigbee is that the hardware is already completely on the market in consumer electronics. Using Bluetooth that is already in devices that workers have available reduces intrusiveness and introduction of new devices [79]. For the network formed of non-worker-related units, the intrusiveness is minimal to non-existent. Nevertheless (local) setups for gateways connected to a wider network or full installations of a local sensor network have effects on maintenance and working procedures.

11. Coverage and Robustness
12. Near LOS communication could be achieved with the Bluetooth [72,82] tests underground. ZigBee has experiments with a focus on underground deployment described alongside others in [83,84,85]. The penetration of rock or even rock masses has not yet been covered by research.
13. Scalability and Number of Users

All technologies can usually function with dozens of users [41]. Scalability with mesh functionality inherent in most ZigBee products is important in dynamic mining environments. Bluetooth does not inherently use such an approach.

14. Data Rate/Network performance
  Bluetooth: 50 Mbits/s [70]

15. ZigBee: 250 Kbits/s [86]

The table below summarizes the technologies and their classification in each evaluation criterion introduced in Section 4. The scales for each criterion consist of three ordered labels, starting with the “worst” and ending with the “best” label. For better recognition, the labels are marked with colours in the Table 2, ranging from red (worst) to green (best) for each category.

### 6.4. Conclusions

In the tracking and tracing area, there are technologies with different advantages and disadvantages. While Bluetooth is a very well-known and successful standard with constant modernisation, ZigBee has a strong manufacturer-provided mesh structure that allows it to be used as local sensor and tracking network, which is also of interest for the mining sector. These use cases are similar to the LUM, just at a more performant and dedicated local level. The flexibility of this network is important because nodes do not need to be purely stationary and can run at low power consumption.

Due to the low infrastructure and high approval, tests and installations are comparatively easy for all technologies. It must therefore ultimately be decided for each individual company which network is considered the most profitable for its technical advantages.

## 7. Long-Range Underground Monitoring

### 7.1. Technology Selection

Long-Range Underground Monitoring applications require long ranges, low power consumption and low cost. These properties can be met by Low Power Wide Area Network (LPWAN) technologies. There are a lot of LPWAN technologies on the market to choose from and the technologies differ in a lot of points. Regarding underground mining operations, the difference between operator-based and private LPWA networks is the most crucial. Operator-based LPWAN networks (e.g., Sigfox or NB-IoT) are maintained and hosted by network carriers, usually in extensive surface networks covering whole regions or countries. For mining operations in dynamic environments, the network infrastructure has to be added, adapted and moved. This task makes it less feasible to have the infrastructure of the network maintained by another party chosen from a very limited list and cannot be done by the mine operator or a wide list of contractors. Furthermore, licensing is limiting the possibility of open and independent research and tests. Therefore, in underground mining operations, privately hosted LPWAN technologies should be preferred. However, in surface mining the usage of operator-based LPWANs is feasible. A hybrid approach of multiple technologies (operator-based and private) can be of an advantage for combined surface and underground operations.

An overview and comparison of current LPWAN technologies regarding technical, implementation and functional factors is given in [10]. With that publication, a first selection and evaluation of LPWAN technologies can be conducted.

When considering only private LPWAN networks, LoRa, Weightless, Wi-SUN, DASH7, IQRF and MIOTY technologies should be considered (see [10]). Evaluation and possible potentials of these technologies regarding mining operations will be derived in this paper.

### 7.2. Technology Description

#### 7.2.1. LoRa

LoRa (long range) and LoRaWAN^®^ describe a low-power wide-area network (LPWAN) protocol used for IoT applications. The term LoRa refers to the proprietary physical layer by Semtech (also called LoRaPHY), and LoRaWAN describes the MAC Layer specification. LoRaWAN^®^ is an open MAC protocol specification which is managed by the LoRa Alliance^®^. The LPWAN operates in ISM sub-GHz frequency bands with data rates between 0.3 to 37.5 Kbps [38,87,88].

LoRa LPWAN is used in Smart City applications with various use-cases in agriculture [89], traffic monitoring [90], environmental monitoring [91], waste management [92], industry process monitoring and energy monitoring [93].

In mining, LoRa technology has successfully been implemented for conveyor belt monitoring. Live sensor data transmitted via LoRa can help to detect rips in the belt and trigger a shut-off [39].

The network consists of three devices present: end-devices, gateways, and network servers. One or multiple gateways handle the received packets from the end devices and requested downlink packets from the network server. The network server connects to IP-based (cloud) applications which process the received data [38,94]. The topology of the devices within the LPWAN network follows a star-of-stars topology with gateways being the central points of the network [88,94]. Due to the topology of the network, dynamic extension of the coverage of the network is done by adding more gateways to the network which need to be connected to a (wired) IP-based network, which limits the ability to extend the network dynamically in quickly changing environments. To overcome the star-of-stars topology limitations not allowing for Device-to-Device communication, [95] summarize recent research findings on multi-hop LoRa networks allowing for extended coverage, improved scalability, network reliability and energy efficiency. [96] propose a LoRa Mesh protocol allowing for device-to-device communication and meshed network architecture. In [97], a linear underground LoRa multi-hop node architecture with an ad-hoc transmission protocol optimizing for node wake-up times with the aim of energy consumption minimization is presented. Similar work on developing a linear multi-hop LoRa network is done in [20].

The focus of this work is on investigating signal strength in underground environments with and without line-of-sight of communication nodes and probabilistic evaluations of successful packet delivery within the multi-hop network.

Ref. [98] investigate transmission ranges of Lora compared to Wi-Fi in underground environments with the result that LoRa has up to 2/3 higher range than Wi-Fi. In [99], LoRa is used in an open-cast mine environment propagating real-time data.

#### 7.2.2. Weightless

Weightless is an open LPWAN protocol which was created by the Weightless Special Interest Group (SIG) (now Weightless Alliance) which was formed by companies such as Neul, Landis+Gyr, Cable & Wireless and ARM [10,100]. Originally, Weightless consisted of three technologies: Weightless-N, -W and -P. Weightless-W was designed to operate in TV whitespace bands and Weightless-N was an uplink-only technology. Weightless-P was a true-bidirectional narrowband technology operating in licensed and unlicensed ISM frequencies. Weightless-P prevailed in the market, and today Weightless simply refers to that technology [100]. The technology offers uplink rates between 0.625 and 100 kbps, uplink data rates between 6.25 and 100 kbps and used time and frequency division multiple access (TDMA and FDMA) and operates on licensed and unlicensed sub-GHz bands (e.g., 470, 510, 840, 868, 902–928 MHz, depending on the operation region) [10,100].

Weightless networks consist of base stations, end devices and backend network management services [101,102]. The network is operated as a private network. The network has a star topology with the base station(s) as central node(s) [103].

The technology is used for smart-metering electric vehicle charging or forecasting solar power generation. A Taiwan Power Company uses the technology to collect 27 million Meter Messages from over 250,000 m daily. The meters are typically in dense urban and underground (basement) environments [102]. In the context of underground mining there is, to the best knowledge of the authors, no publication or known use case. However, the successful application of the technology in smart metering applications (dense urban environment and basement installations) could suggest that the technology is also suitable for some use-cases in the underground mining sector.

#### 7.2.3. Wi-SUN

Wi-SUN is an open standard based on IEEE802, IETD, TIA and ETSI. The physical layer is based on IEEE802.15.4g and allows for data rates between 50 and 300 kbps. Wi-SUN is IP-based and hence interconnects with Ethernet and Wi-Fi [104]. The standard offers mesh network functionality but also a star topology [10].

Wi-SUN technology is used for smart utility networks in smart cities, e.g., advanced metering infrastructure and smart traffic lighting or in smart agriculture application. The city of Miami uses Wi-SUN-enabled devices to connect nearly 500,000 streetlights within a mesh network [104].

To the best knowledge of the authors, use cases in underground mining do not exist; however, the idea of using the technology underground is suggested in [105]. Offering Mesh functionality might be useful for underground application, since the network can be scaled dynamically, which might be beneficial in mining processes in dynamic environments. The technology and its applicability in underground mining is, however, up to future research.

#### 7.2.4. DASH7

DASH7 is a LPWAN protocol specification based on ISO 18000-7 [106] which was originally used for active RFID devices. DASH7 has been modified since and now allows for communication over up to two kilometres. It operates in the 433, 868 and 915 ISM bands and can be applied for data rates between 13 and 200 kbps. The technology is represented through the DASH7 Alliance [107].

The network consists of gateways, nodes and network management software and the topology is a star topology with gateways as central nodes [10,107,108].

The technology is used in applications for smart city, smart parking, safety systems or plants monitoring systems [107]. For underground mining, the use of the technology has been described in [109] for underground event reporting and localization of miners. The authors compare the performance of DASH7 and ZigBee and introduce a multi-hop communication protocol to expand the network in a mining environment. In [110], the link performance of DASH7 in an underground environment is presented. The technology offered ranges of around 100 m in an underground environment. However, more research needs to be conducted to evaluate the performance of DASH7 LPWAN for underground usage.

#### 7.2.5. IQRF

IQRF is a LPWAN wireless mesh technology developed by the IQRF Technology company. The technology operates in 433, 787, 868, 915 and 920 Sub-GHz ISM bands and allows for ranges of tens of meters inside and hundreds of meters in free spaces with a data rate of 19.8 kbps. The coverage can be extended by forming a mesh network with up to 240 devices [10,111,112].

The network consists of communication nodes which can form a mesh network and communicate with each other. Use Cases for IQRF LPWAN technology range from lighting and temperature control, heat and CO_2_ monitoring or wireless torque wrenches in car production processes [10,111,112].

In [113], link performance in line-of-sight (LOS) and non-LOS scenarios in an urban environment were investigated. Signal investigations were undertaken around a house block, and non-LOS scenarios were performed on the adjacent (one turn) or opposite side (two turns) of the block. Measurements and path loss model-based analysis resulted in estimated non-LOS ranges of 660 m (one turn) and 90 m (two turns) when an external antenna in the transceiver was used.

To the best knowledge of the authors, there are no use cases and studies for IQRF usage in underground mining environments. The possibility of forming a mesh network might be useful for usage in dynamic underground mining environments to expand the network dynamically. Further research can evaluate the technologies’ performance for mining.

#### 7.2.6. MIOTY

MIOTY is a LPWAN technology developed by the Fraunhofer Institute IIS, and the technology is standardized by ETSI [114]. It offers data rates of 407 bps and operates in 868 and 915 Sub GHz ISM bands. In rural areas, MIOTY offers ranges of up to 15 km and a range of up to 5 km in urban environments [10,115].

The network consists of gateways, nodes and network management software [116] and has a capacity for 1M+ devices and an uplink capacity of around 1.5 million messages per day per base station [117]. The technology uses Telegram Splitting Multiple Access (TSMA) by transmitting sub-packets over different frequencies and times. A message is split in sub-packets and sent on different frequencies on pseudo-random times and reassembled by the receiver, making the technology less prone to interference [118].

MIOTY technology is used for applications for smart cities, buildings, logistics and agriculture [118]. In underground mining, MIOTY has also been tested, resulting in a 3 km underground range in a tunnel system [37]. The technology is also used for mine climatization control [119] and aimed at further applications of asset tracking, environment monitoring, waste management and predictive maintenance in mining environments [120].

Further research into MIOTY underground use cases is not known, to the best knowledge of the authors, and should be considered for evaluating the technology performance in underground environments.

### 7.3. Classification of Technologies by Parameter

16. Market Maturity/TRL

All considered LPWAN technologies offer products on the market and have been successfully tested in various use cases as described above; however, not all have been tested in underground mining environments. Each technology is therefore at least at the TRL 5–6 level. In terms of underground mining environments, only LoRa, DASH7, and MIOTY have been tested, with LoRa being tested in the most diverse applications compared to the other technologies. DASH7 and MIOTY also were successfully tested in mining environments, but only less independent industry research is available with those technologies. The three technologies therefore are categorized in TRL 7–9.

17. Approval

The considered LPWAN technologies operate in a Sub-GHz ISM band (in Europe, usually 868 MHz). The regulation requires devices transmitting in these frequencies to comply with duty cycling restrictions, which limits the amount of data that can be sent.

18. Required Infrastructure and Intrusiveness

The communication nodes in LPWAN usually have a low energy consumption and can be operated with batteries over a long time. Gateway connections nodes are connected to the network, since they are a bridge for the information from the LPWAN network. These devices are usually externally powered, and network connection is done via an ethernet cable. However, due to the long range, a sparse installation of gateways (compared to technologies like Wi-Fi) is possible.

19. Coverage and Robustness

LPWANs have not been extensively evaluated regarding underground coverage and signalling behaviour. However, in some studies as depicted in Table 3, Near- and/or Non-LOS could be achieved with the LPWAN technologies. The penetration of rock or even rock masses has not been covered by research yet but could potentially be achieved due to the low frequencies used.

20. Scalability and Number of Users

All LPWAN Gateways (star topology) can usually handle hundreds of users (communication nodes). Scalability with mesh functionality is important in dynamic mining environments. LPWAN technologies with built-in mesh technology (Wi-SUN, IQRF) can offer scalability by design. LoRa and MIOTY do not offer built-in mesh functionality; however, there are products (e.g., [121]) that offer mesh functionality for those technologies.

The table below summarizes the technologies and their classification in each evaluation criterion introduced in Section 4. The scales for each criterion consist of three ordered labels, starting with the “worst” and ending with the “best” label. For better recognition, the labels are marked with colours in the Table 3, ranging from red (worst) to green (best) for each category.

### 7.4. Conclusions

LPWAN technologies have great potential in underground mining for Long-Range Underground Mining use cases where robust coverage in wide or dynamic underground environments is needed. They are characterized by a greater range and lower use of network infrastructure but come with lower data throughput.

LPWAN technologies, which can be operated as private networks, are particularly suitable for underground applications. Network infrastructure must be modified, dismantled, or rebuilt in dynamic mining processes, and with privately operated networks this can also be done by the mining operator as opposed to a third party–the network operator.

Ref. [10] gives a good overview of the LPWAN technologies in use today and compares them with respect to different criteria. Private LPWANs suitable for underground use are LoRa, Weightless, Wi-SUN, DASH7, IQRF and MIOTY. LoRa is the LPWAN that has been best studied for underground deployment. LPWAN technologies with integrated mesh functionality–Wi-SUN and IQRF–have been sparsely studied for underground use but offer great potential in terms of scalability in dynamic mining environments due to the mesh functionality. MIOTY offers a potentially better protection from interference in harsh mining environments than the other LPWAN technologies due to the telegram splitting multiple access method. Nonetheless, further research must be conducted to solidify the validity of these technologies in the use cases and environments described.

## 8. Conclusions

WCT are going to play a major role in the advancement of mining operations, since data transmission is the basis for digitalization and automation. Picking the right technology to develop a solution is very difficult due to the variety of WCT available in combination with the possible use case and application.

In this paper, we classified and evaluated wireless communication technologies in terms of their use and potential in the underground mining context. This serves to select the technologies for research and development projects or operational use cases in an advanced mining operation. Except for an all-out installation of a highly performant network throughout the operation (e.g., 5G), which is a cost-intensive option and might even not be possible due to operational constraints, there is no general choice of a WCT as a communication system.

To pick a suitable WCT to develop and implement in an advancing mining operation, use cases and resulting requirements have to be carefully determined in the context of the global operational constraints and harsh environmental conditions. Furthermore, the combination of technologies can be particularly efficient and functional, for example the combination of a local sensing network with a wide-reaching and sparse LPWAN installation to transmit critical information throughout operations or a performant 5G network that is extended by sub-GHz frequencies towards nearby low areas with sensors or users.

A general (wireless) communication infrastructure, that other WCT can leverage on and extend to certain endpoints through gateways can be a valid investment for a mining operation. Here LPWAN technologies have great potential for covering wide areas with minimal installations, when transmitted data can be limited to a minimal extent. Providing connectivity beyond “one network for one purpose”, of course, depends on the overall structure of the mine and its state of automation and digitalization.

## 9. Future Work

Developing and integrating successful use cases of WCT in mining is difficult due to the lack of knowledge in the areas of signal behaviour and transmission, possibilities of sufficient simulation of such and examples as well as suggestions of concrete topological parameters of the network and available evaluations, including quantified results.

For the prediction of signal behaviour, especially in NLOS or penetration use cases, the propagation of signals and their dependence on the geometry as well as the material composition would be very helpful to gain a more general understanding. Further parameters from harsh environment conditions as well as (local) signal noise (e.g., from electric machines) and their effects on the signals have to be evaluated more closely.

This can be achieved through the development of simulations that can incorporate a detailed modelling of the production environment at different process and production stages as well as standardised measurement setups to evaluate performance and determine parameters for such a simulation.

A more practical approach would be the evaluation of potent technologies in valuable use-case setups to generate quantitative results and experience in the use of a WCT. Results from simulations, evaluations and quantitative results could also help to raise the granularity of the proposed technology evaluation criteria for the different use case categories as well. This could also result in a closer guidance regarding what WCT should be used in what environment in combination with the intended use case, enabling the integration of advanced mining technologies for the mining operations of the future.

## Figures and Tables

**Table 1:** Summarized Comparison and Evaluation of Wi-Fi, LTE/4G and 5G.

	Market Maturity	Approval	Req. Infrastructure & Intrusiveness	Coverage and Robustness	Scalability and Number of Users	Data Rate/Network Performance
Wi-Fi 6 GHz	3	3	1	1	3	3+
Wi-Fi 2.4 GHz	3	3	1	1–2	2	2
5G	2	2	1	1–2	3+	3+
LTE 4G	3	1–3	1+	2+	2	2

**Table 2:** Summarized Comparison and Evaluation of Bluetooth and ZigBee.

	Market Maturity	Approval	Req. Infrastructure & Intrusiveness	Coverage and Robustness	Scalability and Number of Users	Data Rate/Network Performance
Bluetooth	3	3	3	1	2	2
ZigBee	2	3	3	2~	2	1

**Table 3:** Summarized Comparison and Evaluation of LoRa, Weightless, Wi-SUN, DASH7, IQRF and Mioty.

	Market Maturity	Approval	Req. Infrastructure & Intrusiveness	Coverage and Robustness	Scalability and Number of Users	Data Rate h/Network Performance
LoRa	3	3	3 (nodes), 2 (gateways)	2–3 [99]	2–3	1 (bps-kbps)
Weightless	2	3	3 (nodes), 2 (gateways)	Not known	2	1 (up to 100 kbps) [100]
Wi-SUN	2	3	3 (nodes), 2 (gateways)	Not known	3	1 (300 kbps) [104]
DASH7	3	3	3 (nodes), 2 (gateways)	2–3 [110]	2	1 (9.6–166 kb/s) [122]
IQRF	2	3	3 (nodes), 2 (gateways)	2 [113]	3	1 (19.8 kb/s) [123]
MIOTY	3	3	3 (nodes), 2 (gateways)	2–3 [37]	2–3	1 (407 bit/s) [119]

## Data Availability

Not applicable.
